# TMS Seeded Diffusion Tensor Imaging Tractography Predicts Permanent Neurological Deficits

**DOI:** 10.3390/cancers14020340

**Published:** 2022-01-11

**Authors:** Matthew Muir, Sarah Prinsloo, Hayley Michener, Jeffrey I. Traylor, Rajan Patel, Ron Gadot, Dhiego Chaves de Almeida Bastos, Vinodh A. Kumar, Sherise Ferguson, Sujit S. Prabhu

**Affiliations:** 1 Department of Neurosurgery, The University of Texas MD Anderson Cancer Center, Houston, TX 77030, USA; mmuir@mdanderson.org (M.M.); sprinsloo@mdanderson.org (S.P.); hkburgess@mdanderson.org (H.M.); ron.gadot@bcm.edu (R.G.); sdferguson@mdanderson.org (S.F.); 2 Department of Neurological Surgery, The University of Texas Southwestern Medical Center, Dallas, TX 77030, USA; jeffrey.traylor@utsouthwestern.edu; 3 Department of Neurosurgery, Baylor College of Medicine, Houston, TX 77030, USA; rajan.patel@bcm.edu; 4 Department of Neurosurgery, Cleveland Clinic, Cleveland, OH 61349, USA; bastosd@ccf.org; 5 Department of Neuroradiology, The University of Texas MD Anderson Cancer Center, Houston, TX 77030, USA; vakumar@mdanderson.org

**Keywords:** glioma, eloquence, neurological deficit, onco-functional balance, functional imaging, transcranial magnetic stimulation, diffusion tensor imaging, tractography

## Abstract

**Simple Summary:**

For brain tumor patients, surgeons must resect as much of the tumor as possible while preserving the patient’s function and quality of life. This requires preoperative imaging that accurately identifies important parts of the brain. Transcranial magnetic stimulation is a way of preoperatively finding the areas of the brain connected to motor function. However, few studies have investigated the accuracy and clinical relevance of the data. In this study, we examine the functional outcomes of patients who had TMS points resected and patients who did not. We aim to address key technical barriers to performing this analysis. We also aim to discern the appropriate role of TMS tractography in preoperative diagnostic imaging. Insights gained from this study can be used to select the right patients and plan for the optimal surgeries.

**Abstract:**

Surgeons must optimize the onco-functional balance by maximizing the extent of resection and minimizing postoperative neurological morbidity. Optimal patient selection and surgical planning requires preoperative identification of nonresectable structures. Transcranial magnetic stimulation is a method of noninvasively mapping the cortical representations of the speech and motor systems. Despite recent promising data, its clinical relevance and appropriate role in a comprehensive mapping approach remains unknown. In this study, we aim to provide direct evidence regarding the clinical utility of transcranial magnetic stimulation by interrogating the eloquence of TMS points. Forty-two glioma patients were included in this retrospective study. We collected motor function outcomes 3 months postoperatively. We overlayed the postoperative MRI onto the preoperative MRI to visualize preoperative TMS points in the context of the surgical cavity. We then generated diffusion tensor imaging tractography to identify meaningful subsets of TMS points. We correlated the resection of preoperative imaging features with clinical outcomes. The resection of TMS-positive points was significantly predictive of permanent deficits (*p* = 0.05). However, four out of eight patients had TMS-positive points resected without a permanent deficit. DTI tractography at a 75% FA threshold identified which TMS points are essential and which are amenable to surgical resection. TMS combined with DTI tractography shows a significant prediction of postoperative neurological deficits with both a high positive predictive value and negative predictive value.

## 1. Introduction

Many studies have established a link between the extent of resection and survival in patients with gliomas [1]. However, peri-eloquent tumors represent a unique challenge. Surgeons must balance maximizing the extent of resection with preserving functionality. Studies have shown that permanent postoperative neurological deficits lead to decreased survival in addition to severely decreased quality of life [2]. Traditionally, surgeons have used anatomic factors to preoperatively evaluate eloquence, choose surgical candidates, and plan for surgery. However, accumulating data show that anatomic considerations alone cannot predict functionality [3]. Studies have shown broad inter-individual variability of functional distribution throughout the brain [4]. Additionally, various reports have shown evidence of plastic reorganization of functional cortex in tumor patients, a phenomenon not observed in healthy controls [5,6,7,8]. These data implicate the need to develop preoperative functional mapping modalities.

Transcranial magnetic stimulation (TMS) has emerged as a non-invasive method of mapping functional cortex. However, due to the limited number of TMS devices in neurosurgical centers, scarce data exist validating the clinical utility of this technology. To date, studies have shown varying degrees of correlation of TMS with intraoperative, direct cortical stimulation, between 2 mm and 16 mm [9]. Other studies have shown improved rates of gross total resection (GTR) in cohorts who had preoperative TMS vs. cohorts who did not [10]. However, little data exist that directly investigate the functional relevance of TMS data with respect to clinical outcome [11]. In this study, we investigate the relationship between TMS functional data and permanent neurological motor deficits. We use preoperative and postoperative MRI overlays, termed “perioperative overlays”, to directly study the clinical consequences of resecting cortical and subcortical structures identified using preoperative TMS data. We aim to provide more insight into the clinical relevance of TMS for presurgical motor mapping.

## 2. Materials and Methods

Forty-two patients were included in this retrospective study with the following inclusion criteria: patients aged over 18 with motor eloquent gliomas as determined by preoperative MRI who underwent presurgical TMS motor mapping as well as intraoperative cortical and subcortical mapping (Table 1). We used the following exclusion criteria: patients with postoperative acute infarctions, postoperative disease recurrence in eloquent cortex before a 3-month follow up, and significant postoperative edema causing midline shift >10 mm present at a 3-month follow up. We collected demographic data from electronic medical records as well as clinical outcome data, with the primary outcome being new or worsened postoperative motor deficit present at the 3-month follow up, termed “permanent deficit”. For patients with no preoperative weakness, no deficit was recorded when motor strength was 5/5 for all extremities upon neurological examination by the neurosurgical team. A deficit was recorded when motor strength was <5/5. For patients with preoperative weakness, no deficit was recorded when the neurological exam was the same or better. A deficit was recorded when the exam was one point or more worse. This study was approved by the University of Texas MD Anderson Cancer Center Institutional Review Board (# 2021-0856).

### 2.1. Transcranial Magnetic Stimulation

A navigated transcranial magnetic stimulation (TMS) system was utilized in the present study (NBS System 3.2, Nexstim, Helsinki, Finland). The most likely location of the hand knob was identified anatomically. This area was then stimulated in a random pattern while systematically varying the rotation, tilt and yaw of the magnetic field. The location of maximal motor evoked potential (MEP) was identified. Resting motor threshold (RMT) was identified using this position [1]. MEPs for generating the RMT were measured in the abductor digiti minimi. TMS stimulation was delivered over the primary motor cortex (MI) via a figure-of-eight head coi, at 110% intensity of the resting motor threshold. For quantification of the TMS effect, MEPs were measured in the upper and lower extremity. Positive sites were marked in the 3D brain surface as white dots and this information was sent as a report to the surgeon. These sites were also exported in DICOM files that were to be uploaded to the Neuronavigation system (Elements, BrainLab, Munich, Germany).

### 2.2. Diffusion Tensor Imaging

**Figure 1:**
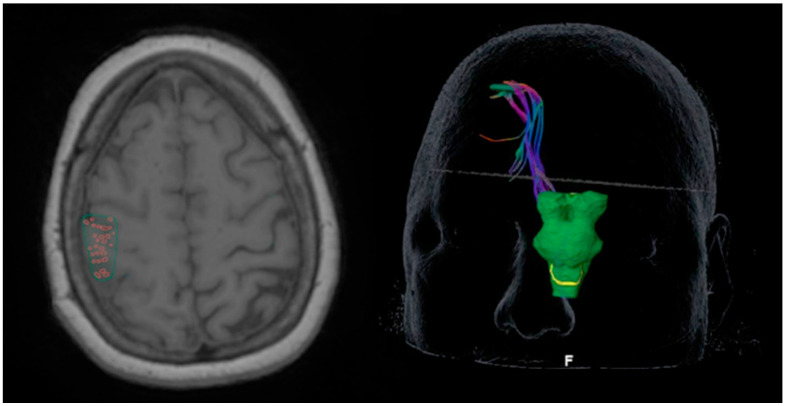
Axial registration T1-weighted registration image illustrates TMS points as the cortical ROI (**left**) and 3D reconstruction corticospinal tract (CST) generated from the TMS seeded tractography (**right**).

DTI and structural MR imaging were performed using a 3T MRI scanner (GE Healthcare, Waukesha, Wisconsin) with an eight-channel head coil. DTI was performed using a diffusion-weighted spin-echo echo-planar imaging sequence (repetition time/echo time = 10,000/120 ms, matrix size = 128 × 128, field of view = 22 × 22 cm^2^, slice thickness = 2.5 mm with no intersection gap, number of diffusion-weighting directions = 32, b value = 1000 s/mm^2^). In total, 44 slices were acquired, covering the medulla to the top of the brain. High-resolution 3D spoiled gradient-echo T1-weighted sequences were acquired for anatomic reference. DTI fiber tractography was generated using a deterministic method (Brainlab Elements, Munich, Germany) based on two regions of interest (ROIs) placed on the fractional anisotropy map. Thresholds of a maximum angulation = 30 and a minimal fiber length = 110 were applied for the fiber tracking. We used the TMS-positive points as the cortical region-of-interest (ROI) and the brainstem for the subcortical ROI. We used the following parameters for DTI tractography, outlined in a previous study [12]. The minimum fiber length of 110 mm and maximum angulation of 30 degrees were preset and were the same across all patients, while we used a standardized FA threshold approach that resulted in a different absolute FA threshold value for each patient. For each patient, we progressively increased the FA threshold until no fibers were generated. We denoted this as the 100% FA threshold for that patient. We generated tracts at 25%, 50%, and 75% for each patient. As a result, the FA threshold percentages were consistent across the entire cohort, while the individual FA threshold varied. Figure 1 shows the objects created from TMS points as well as a 3D reconstruction of TMS-seeded tractography.

### 2.3. Perioperative Overlays

**Figure 2:**
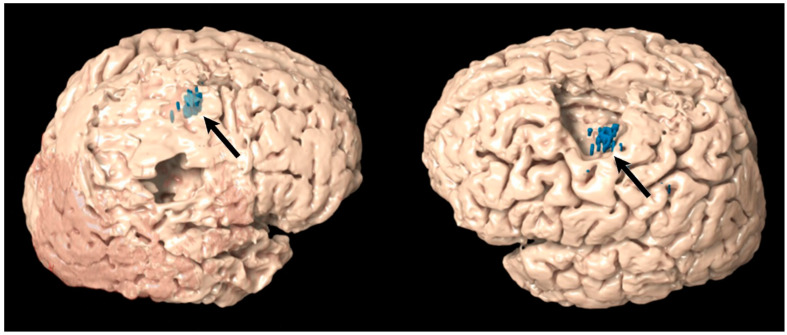
3D reconstruction of TMS points overlayed onto the postoperative resection cavity. Image on left shows a patient with no TMS points resected, while the image on the right shows a patient with several TMS points resected. Arrow points to the TMS points (blue).

We used Brainlab Elements (Brainlab Inc., Munich, Germany) to import and view the Navigated Brain Scan (NBS) data. Objects of the TMS points were created. We analyzed preoperative imaging features, collecting patients with TMS points within the glioma as well as patients with glioma infiltrating the precentral gyrus. We then fused the NBS scan with the preoperative T1 MRI so that the TMS objects could be viewed on the T1 MRI. We then used a semi-elastic fusion approach (Brainlab Elements, Munich, Germany) to superimpose the postoperative MRI onto the preoperative MRI to view preoperative functional and anatomic imaging features in context of the resection cavity. The postoperative MRI was performed the day after surgery for all patients. The fusion was based on co-registrations using intra-axial markers determined by the Brainlab Elements algorithm. Figure 2 shows a 3D reconstruction of the precentral gyrus and TMS points overlayed onto the resection cavity.

### 2.4. Statistical Analysis

We used SPSS (IBM Corp, Armonk, NY, USA) for our statistical analysis. We constructed a binary classifier system using TMS variables to predict permanent deficits. We performed univariate binary logistic regression to find significant predictors of permanent deficits. We derived odds ratios and 95% confidence intervals with binary logistic regression and evaluated statistical significance using the Fisher’s exact test at a significance level of *p* < 0.05. We modeled the predictive value of resection versus preservation of white matter tracts (WMTs) identified at various FA thresholds with contingency tables. A true positive was defined as the site of a TMS positive point and/or WMTs resected with a corresponding permanent deficit. A false positive was defined as the site resected with no corresponding permanent deficit. A true negative was defined as the site preserved with no permanent deficit. A false negative was defined as the site preserved with a permanent deficit. We calculated the positive predictive value (PPV) and negative predictive value (NPV) for resection of WMTs identified at various FA thresholds.

## 3. Results

Table 1 shows preoperative, intraoperative, and postoperative patient characteristics.

**Table 1:** Patient characteristics.

Number	Age	Sex	Histology	Preoperative Weakness	TMS Points Displaced from Precentral Gyrus	TMS Positive Points within Tumor	Tumor Infiltration of Precentral Gyrus	TMS Positive Point Resection	Resection of Precentral Gyrus	New or Worsened Permanent Deficit
1	64	Male	Anaplastic ependymoma	Yes	No	No	Yes	No	Yes	No
2	54	Female	Grade II oligodendroglioma	No	No	No	No	No	No	No
3	43	Female	Diffuse astrocytoma	No	Yes	Yes	Yes	Yes	No	No
4	35	Male	Diffuse astrocytoma	No	No	No	No	No	No	No
5	62	Female	GBM	Yes	Yes	Yes	Yes	Yes	Yes	Yes
6	35	Male	Anaplastic oligodendroglioma	No	Yes	No	No	No	No	No
7	65	Female	GBM	No	No	No	Yes	No	Yes	No
8	66	Male	GBM	No	Yes	Yes	Yes	Yes	Yes	Yes
9	72	Female	GBM	Yes	Yes	Yes	Yes	No	Yes	No
10	59	Male	GBM	No	Yes	No	No	No	Yes	Yes
11	45	Male	GBM	No	Yes	No	Yes	Yes	Yes	Yes
12	30	Male	Grade II oligodendroglioma	No	Yes	Yes	Yes	No	Yes	No
13	55	Female	GBM	No	No	No	No	No	Yes	No
14	68	Male	GBM	No	Yes	No	Yes	No	Yes	No
15	40	Male	Grade II oligodendroglioma	No	Yes	No	Yes	Yes	No	No
16	30	Male	Diffuse astrocytoma	No	Yes	Yes	Yes	No	No	No
17	36	Male	GBM	No	No	No	Yes	No	No	Yes
18	34	Female	Anaplastic astrocytoma	No	Yes	Yes	Yes	Yes	Yes	Yes
19	59	Male	Diffuse astrocytoma	No	Yes	No	Yes	No	Yes	No
20	40	Male	GBM	No	No	No	Yes	No	Yes	No
21	59	Male	GBM	Yes	No	No	No	No	No	No
22	57	Female	GBM	Yes	Yes	No	Yes	No	No	No
23	33	Male	Diffuse astrocytoma	No	Yes	Yes	Yes	No	No	No
24	36	Male	Anaplastic oligodendroglioma	No	No	No	No	No	Yes	No
25	39	Male	GBM	Yes	Yes	No	No	No	No	No
26	53	Female	Grade II oligodendroglioma	No	No	No	Yes	No	No	No
27	56	Female	Grade II oligodendroglioma	No	No	No	No	No	No	No
28	71	Male	GBM	No	No	No	Yes	No	No	No
29	48	Male	Grade II oligodendroglioma	No	No	No	No	No	No	No
30	42	Male	Ependymoma	Yes	Yes	No	Yes	No	Yes	No
31	46	Female	Anaplastic astrocytoma	No	No	No	Yes	No	No	No
32	67	Female	GBM	No	No	No	No	No	No	No
33	73	Female	Diffuse astrocytoma	No	No	No	Yes	No	Yes	Yes
34	37	Female	Grade II oligodendroglioma	Yes	No	No	No	No	No	No
35	34	Male	Diffuse astrocytoma	No	No	No	No	No	No	No
36	77	Male	GBM	No	No	No	No	No	Yes	No
37	64	Male	GBM	Yes	No	No	Yes	No	Yes	No
38	61	Male	GBM	No	No	Yes	Yes	Yes	Yes	No
39	38	Male	Anaplastic oligodendroglioma	No	Yes	No	Yes	Yes	No	No
40	51	Female	GBM	Yes	No	No	No	No	No	No
41	47	Male	Diffuse astrocytoma	Yes	Yes	No	Yes	No	No	No
42	65	Male	Anaplastic astrocytoma	Yes	No	No	No	No	No	No

Nine patients (21%) had TMS-positive points within the tumor. Eight patients (19%) had TMS-positive points resected. Seven patients (17%) had a new or worsened postoperative neurological deficit present at the 3-month follow up. Table 2 summarizes univariate logistic regression analysis for perioperative TMS features.

Out of nine patients with TMS-positive points within the tumor, three had permanent deficits (33%). Out of eight patients with TMS positive points resected, four had permanent deficits (50%). TMS-positive points within the glioma were not significantly predictive of permanent neurological deficits using a logistic regression model. However, resection of TMS positive points was significantly predictive of permanent deficits (*p* = 0.004).

**Figure 3:**
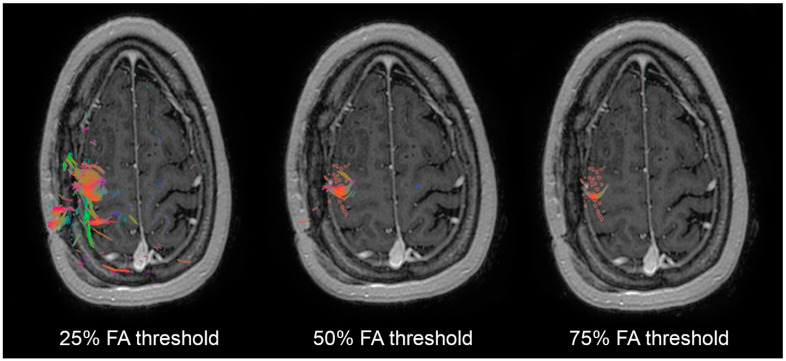
Effect of varying fractional anisotropic (FA) thresholds using TMS as the cortical ROI to generate the corticospinal tract. Left depicts white matter tracks identified by a 25% FA threshold, middle depicts a 50% FA threshold, and right depicts a 75% FA threshold.

We then generated tractography using TMS points for the cortical ROI with FA thresholds of 25%, 50%, and 75%. Figure 3 shows the result of each of these FA thresholds, illustrating that a 25% threshold identifies a maximal amount of white matter tracts (WMT) connecting to the entire TMS cortex.

A 75% FA threshold shows only a small subset of the TMS points with connecting WMT. We used contingency tables to model the predictive value of the resection of WMTs identified by FA thresholds at 25%, 50%, and 75% (Table 3).

The PPV for the 25% FA threshold was 29% and the NPV was 97%. The PPV for the 50% FA threshold was 50% and the NPV was 97%. The PPV for the 75% threshold was 87% and the NPV was 97%. We used binary logistic regression to find that the resection of WMTs identified at a 25% FA threshold was not significantly predictive of permanent deficits. Resections at 50% (OR = 29, *p* = 0.004) and 75% (OR = 204, *p* ≤ 0.0001) were significantly predictive of permanent deficits. Table 4 shows statistical variables for each FA threshold in relation to permanent deficits.

**Figure 4:**
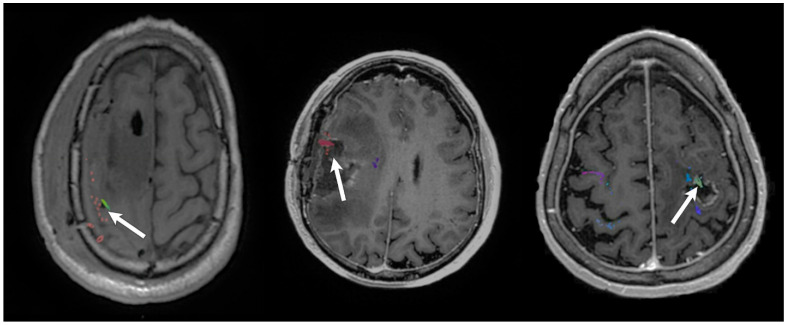
Three patients with permanent deficits showing the resection cavity overlapping with white matter tracts identified by TMS-seeded DTI tractography at 75% FA threshold. Arrow points to the overlap indicating resection of tracts.

Figure 4 illustrates three cases of patients with white matter tracts identified by TMS tractography at a 75% FA threshold resected.

**Figure 5:**
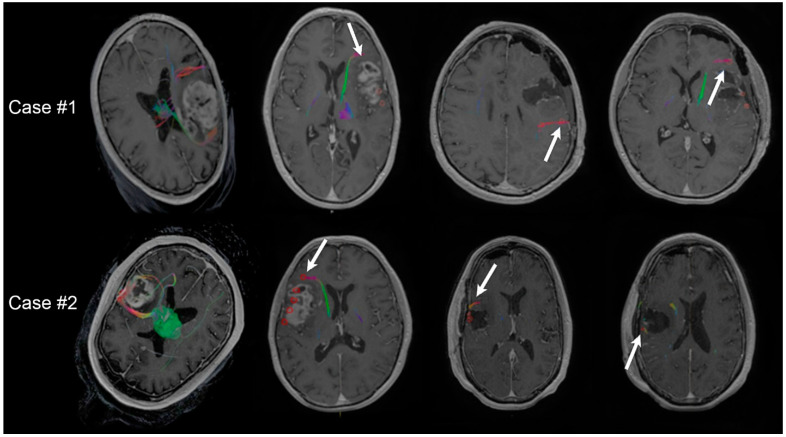
Case illustration of two patients without permanent motor deficits. Left picture shows TMS-seeded tractography at 75% FA threshold identifying white matter tracts splitting around the lesions. Positive TMS points spanned the entire lesions; however, white matter tracts only connected to the edges of the functional cortex. The resection removed the bulk of the TMS-positive cortex yet preserved the edges with connecting white matter tracts. The patient made a complete neurological recovery. Arrows indicate DTI tractography WMT connections to TMS points.

These images show the overlay of the postoperative resection cavity with the preoperative TMS DTI tractography data. All three patients exhibited permanent motor deficits. Figure 5 illustrates two cases of patients with TMS positive points spanning the entire length of the tumor.

However, WMTs identified by a 75% FA threshold diverged and split around the tumor, connecting at the edges of the TMS cortex. The resection removed the bulk of the TMS-positive cortex yet preserved the edges of the cortex that had connecting WMTs. These patients made a full neurological recovery.

## 4. Discussion

While many studies have investigated the correlation between preoperative TMS points and intraoperative DCS mapping, only one study has analyzed the clinical result of resecting TMS points. Moser et al. found an association between resection of TMS positive prerolandic points and permanent neurological deficits [11]. To build upon this study, we provided evidence from our own cohort and further refined the methodology. Retrospectively overlaying postoperative MRI onto the preoperative MRI (“perioperative overlay”) allows the analysis of various resections. However, technical limitations such as distorted overlays, ambiguous clinical outcomes, and confounding factors can undermine confidence in the data. We pursued key methods to overcome these challenges and outline them below.

Previously, our group encountered challenges attempting to study TMS data using overlays of preoperative and postoperative MRIs. For easy and accurate segmentation of TMS points to allow for 3D reconstruction, we previously used a DICOM image series with only the nose, ears, and TMS-positive points. Performing co-registrations using only these extra-axial landmarks led to significant overlay distortion and inaccurate fusions. To address these issues, we pursued more sophisticated methods of segmenting TMS points to allow subsequent overlays using a DICOM image with TMS points in context of the entire preoperative MRI. This fusion was based on co-registrations using intra-axial markers and significantly reduced the overlay distortion. Additionally, we used a semi-elastic fusion approach for correcting spatial inaccuracies from geometric image distortions in the overlays. This distortion correction algorithm has previously been shown to significantly increase the accuracy of image fusions for tractography reconstruction [13]. Our group applied this algorithm specifically to address image distortion in pre- and postoperative MRI overlays resulting from brain shifts occurring during the perioperative course. This two-pronged approach significantly improved the fidelity of our image overlays and allowed for direct investigations of long-term clinical outcomes resulting from the resection of TMS identified structures.

Additionally, a major methodological factor in our study was selecting neurological status at the 3-month follow up as the main outcome. Many patients exhibit transient postoperative deficits that eventually subside as edema decreases or plastic reorganization occurs. Eleven patients in our cohort had postoperative deficits present at the 1-month follow up, while only seven of those patients had persistent deficits through the 3-month follow up. We selected the 3-month follow up for the primary outcome to capture the most meaningful clinical result: true permanent deficits instead of transient. We also systematically excluded patients with postoperative sequala that could also cause neurological deficits, such as significant edema causing a midline shift greater than 10 mm present at 3 month follow up, disease recurrence within eloquent cortex within the first 3 months postop, or a postoperative acute infarction. A midline shift of 10 mm or greater was chosen because studies have shown that midline shifts above this threshold produce neurological deficits [14,15]. These exclusion criteria were an attempt to mitigate confounding factors. In our view, each of these methodological approaches, from the sophisticated overlays to the primary outcome to the exclusion criteria, are crucial to directly analyze the most relevant clinical result of various resections in order to provide accurate predictors of eloquent tissue for patient selection and surgical planning.

We found that TMS points within the tumor did not significantly predict permanent deficits, while the resection of TMS points did. We then noted that despite these results, four out of eight patients with TMS positive points resected had no permanent deficits. We defined properties of TMS points that explained this discrepancy and rendered them relevant to functional outcome. Because of numerous reports detailing limited subcortical plasticity and the importance of sparing subcortical white matter tracts [16,17,18], we hypothesized that tractography could delineate between indispensable TMS points and TMS points that can be safely resected.

Using standardized fractional anisotropic (FA) threshold values previously described [12], we generated DTI tractography using the TMS points as the cortical ROI. For an exploratory analysis, we initially generated tracts using FA thresholds at 25%, 50%, and 75%.

To determine which FA threshold reveals the most clinically relevant WMTs, we overlayed the postoperative resection cavity onto the preoperative MRI containing the tractography at each FA threshold and correlated this with clinical outcome. We constructed a binary classifier system from these data and used contingency tables to model the predictive value of resection of WMTs identified at each FA threshold, shown in Table 3.

The PPV progressively increased as the FA increased, while the NPV was 97% across all three FA thresholds. The 75% FA threshold showed the highest PPV at 87%. Logistic regression showed that predictive models of 25% FA thresholds were not statistically significant, while models at 50% and 75% FA thresholds were statistically significant. The 75% FA threshold model showed the highest OR (204) and lowest *p*-value (<0.0001). These results showed that tractography generated at 75% FA thresholds provides the strongest predictor of permanent deficits, indicating the identification of true eloquent, non-resectable tissue.

Previous studies on TMS tractography have reached a variety of conclusions regarding the optimal FA threshold, sometimes stratified by motor-associated versus language-associated WMTs. Frey et al. initially found that surgeons rated the WMTs identified at a 75% FA threshold as more surgically useful compared to a 50% FA threshold for corticospinal tract (CST) mapping [12]. However, Sollman et al. found that FA thresholds at 75% do not identify all the relevant language fibers, outlining the need for tracking performed at 25% or 50% FA threshold for patients with language eloquent tumors [19]. Another large study found significant correlations in preoperative lesion-to-tract distances (LTDs) and functional outcome. The authors found that LTDs for the CST showed significant correlations to functional outcome using FA thresholds at 25%, 50%, and 75% [20]. However, they also found that LTDs for the arcuate fasciculus were only significantly correlated with functional outcome at a 25% FA threshold [20]. Future research should focus on delineating the optimal FA threshold for specific tracts.

Numerous reports detail the importance of preserving axonal connectivity by sparing subcortical white matter tracts to allow for postoperative functional compensation [16,17,18]. However, data from our study show that some white matter tracts can be safely resected while others are indispensable. WMTs identified at lower FA thresholds were safely resected without permanent sequela. However, resection of WMTs identified by a 75% FA threshold showed excellent positive predictive value and negative predictive value for permanent deficits. While mechanistic elucidation requires further studies, a possible hypothesis is that the higher FA thresholds identify the most *active* WMTs. Increased neural activity leads to increased myelination, which has been shown to increase fractional anisotropy [21]. Gliomas cause dramatic cortical reorganization, leading to a topographic displacement of active cortical hubs, which then recruit local WMTs for their specific function [22]. Perhaps the higher FA thresholds identify active WMTs, while the lower FA thresholds identify latent WMTs, recently inactive due to cortical reorganization to a distant site. Regardless of the mechanism, this study provides a method for identifying a subset of WMTs that can be safely resected without permanent neurological deficits.

Many other studies have attempted to preoperatively define eloquence and resectability using a variety of methods. Lus et al. constructed an atlas using 58 patients undergoing glioma resections while using intraoperative mapping techniques. They aggregated and spatially normalized postoperative MRIs into the Montreal Neurological Institute (MNI) space to determine which anatomic regions consistently display residual tumor. The authors derived the probability of resection for each anatomic region across the entire cohort. The result was an atlas that assigned probabilities of a safe resection for each anatomic region for preoperative patient selection and surgical planning [18]. Muller et al. used a similar methodology, denoting these atlases as “resection probability maps”. The authors constructed a resection probability map from a cohort of 915 patients and showed that it predicts resectability and survival better than anatomic eloquence grading [23]. Additionally, a multitude of other methods for determining eloquence and resectability have been evaluated. Structural MRI, task-based functional MRI, resting-state functional MRI, magnetoencephalography, and white matter tractography have all been evaluated for preoperative identification of eloquent tissue by comparisons to intraoperative DCS [24,25,26,27].

However, conclusions from these studies reflect the limitations of intraoperative mapping data. Many studies have found that intraoperative mapping improves patient outcomes [28]. However, no studies directly investigate the functional consequence of resecting DCS points. Multiple studies have shown a significant correlation of TMS-positive points with intraoperative DCS points [9]. Additionally, data from our study show that a subset of TMS points can be safely resected without permanent sequela. Perhaps a subset of DCS points amenable to safe resection could be identified, although ethical constraints pose a significant challenge to testing this hypothesis. Regardless, these findings call into question the use of intraoperative mapping data as the gold standard for evaluating preoperative imaging modalities. In our view, clinical outcomes should be utilized to capture the most relevant corollary to preoperative imaging features.

Other studies have used functional outcome to evaluate eloquence and resectability; however, these studies correlate preoperative LTDs to the outcome [29]. Few have controlled for variations in the resection by using pre- and postoperative MRI overlays [30]. Our preliminary data found no significant correlations between preoperative imaging markers and functional outcomes, prompting further analysis accounting for intersurgical variations. In our cohort, three patients showed TMS-positive points within the tumor but did not undergo a corresponding resection of TMS points. Additionally, three patients did not show TMS-positive points within the tumor but did have TMS-positive points resected. These results illustrate the importance of controlling for surgical variation. Alternative approaches between surgeons as well as between cases could introduce a significant confounder to the analysis. Perhaps additional studies using long-term functional outcome measures combined with surgical cavity analysis using perioperative overlays could provide more robust data for drawing clinically relevant conclusions regarding preoperative imaging modalities.

Only two patients in our cohort deviated from the predictive paradigm with a 75% FA threshold: one false positive and one false negative. The overlay for the false-positive patient showed TMS points multiple millimeters superficial to the cortex on the postoperative MRI, evidence of significant overlay distortion due to brain shift. Further isolated image analysis revealed that the relevant WMTs were 36 mm deep on the preoperative MRI, while the resection was only 32 mm deep on the postoperative MRI, indicating probable WMT preservation. Most likely, the cortical deformation distorted the overlay, falsely identifying the WMTs from the preoperative MRI within the resection cavity on the postoperative MRI. Additionally, the patient who was a false negative showed persistent deficits through the 3-month follow up; however, at the 1-year follow up, the deficit had resolved, indicating that the plastic reorganization process in this patient took more time. Excluding these patients with confounding factors increased both the PPV and NPV to 100% for the 75% FA threshold for TMS tractography.

We also performed the same analysis on a separate cohort of metastases patients but did not find similar results. We observed significantly more overlay distortion due to brain shift. Compared to gliomas, we hypothesized that metastases produce significantly greater brain shift and tissue deformation due to mass effect. Metastases compress native tissue, while gliomas infiltrate. This mechanistic difference could prohibit meaningful analysis of metastases from pre- and postoperative MRI overlays, while infiltrative glioma cohorts do not experience the same limitations. However, despite the technical limitations and lack of direct evidence for metastases cohorts, perhaps the same conclusions could extrapolate to these patients. Future research should pursue further methods of addressing overlay distortion due to brain shift, as well as methods for quantifying brain shift.

Future research should also pursue more consistent long-term patient follow up to avoid false negatives and further refine the primary outcome to capture the most relevant clinical result. Analyses should be extended to cohorts with language eloquent lesions in hope of identifying similar metrics for patient selection and surgical planning. TMS-seeded DTI tractography should also be compared in a similar manner to other preoperative imaging modalities, such as functional MRI and anatomic-seeded DTI tractography. Additionally, despite the significant results from this study, DTI has well-documented drawbacks [31,32]. Future research should combine TMS seeding with methods for high-definition fiber tractography, such as Q-ball or constrained spherical deconvolution [33,34].

## 5. Conclusions

The resection of TMS points is significantly predictive of permanent neurological deficits. The resection of white matter tracks identified by TMS-seeded tractography specifically with an FA threshold of 75% leads to permanent motor impairment. Tractography with this FA threshold delineates which TMS points are essential and which are amenable to surgical resection. TMS combined with DTI tractography shows significant prediction of postoperative neurological deficits with both a high positive predictive value and a negative predictive value. This imaging combination shows great potential for optimizing the onco-functional balance and reducing postoperative permanent neurological impairments while maximizing the extent of resection.

## Figures and Tables

**Table 2:** Univariate binary logistic regression for predication of permanent deficit from cortical TMS perioperative variables.

	No. of Patients	Permanent Deficits, No. (%)	OR	95% CI	*p* Value
TMS positive points within tumor					
Yes	9	3 (33)	3.6	0.64–20.57	0.15
No	33	4 (12)			
TMS positive points resection					
Yes	8	4 (50)	10.3	1.67–64.00	0.012
No	34	3 (8.8)			

**Table 3:** Contingency tables showing the predictive value of resection vs. preservation of WMTs at difference FA thresholds.

Tractography at 25% FA	Deficit	No Deficit	Tractography at 50% FA	Deficit	No Deficit	Tractography at 75% FA	Deficit	No Deficit
Resection	6	15	Resection	6	6	Resection	6	1
Preservation	1	29	Preservation	1	29	Preservation	1	34

**Table 4:** Univariate binary logistic regression showing the predictive value of resection of TMS identified WMTs at various fractional anisotropic thresholds (FATs).

	No. of Patients	Permanent Deficits, No. (%)	OR	95% CI	*p* Value
Resection of 25% FAT TMS WMTs					
Yes	21	6 (29%)	8	0.87–73.68	0.066
No	21	1 (4.8%)			
Resection of 50% FAT TMS WMTs					
Yes	12	6 (50%)	29	2.93–287.02	0.004
No	29	1 (3.4%)			
Resection of 75% FAT TMS WMTs					
Yes	7	6 (86%)	204	11.17– 3724.26	<0.0001
No	35	1 (2.9%)			

## Data Availability

The data presented in this study are available in the article.
